# Forced Sexual Experiences as Risk Factor for Self-Reported HIV Infection among Southern African Lesbian and Bisexual Women

**DOI:** 10.1371/journal.pone.0053552

**Published:** 2013-01-09

**Authors:** Theo G. M. Sandfort, Linda R. M. Baumann, Zethu Matebeni, Vasu Reddy, Ian Southey-Swartz

**Affiliations:** 1 HIV Center for Clinical and Behavioral Studies, New York State Psychiatric Institute and Columbia University, New York, New York, United States of America; 2 Out-Right Namibia, Windhoek, Namibia; 3 Institute for Humanities in Africa, University of Cape Town, Cape Town, South Africa; 4 Human Sciences Research Council, Human and Social Development Programme, Pretoria, South Africa; 5 University of KwaZulu-Natal, Durban, South Africa; 6 Open Society Initiative for Southern Africa, Johannesburg, South Africa; Rollins School of Public Health, Emory University, United States of America

## Abstract

Even though women who have sex with women are usually understood to be at no or very low risk for HIV infection, we explored whether lesbian and bisexual women in a geographical area with high HIV prevalence (Southern Africa) get tested for HIV and whether, among those women who get tested, there are women who live with HIV/AIDS. The study was conducted in collaboration with community-based organizations in Botswana, Namibia, South Africa and Zimbabwe. Data were collected via written surveys of women who in the preceding year had had sex with a woman (18 years and older; N = 591). Most participating women identified as lesbian and black. Almost half of the women (47.2%) reported ever having had consensual heterosexual sex. Engagement in transactional sex (lifetime) was reported by 18.6% of all women. Forced sex by men or women was reported by 31.1% of all women. A large proportion of the women reported to ever have been tested for HIV (78.3%); number of lifetime female and male partners was independently associated with having been tested; women who had engaged in transactional sex with women only or with women and men were less likely to have been tested. Self-reported HIV prevalence among tested women who knew their serostatus was 9.6%. Besides age, the sole independent predictor of a positive serostatus was having experienced forced sex by men, by women, or by both men and women. Study findings indicate that despite the image of invulnerability, HIV/AIDS is a reality for lesbian and bisexual women in Southern Africa. Surprisingly, it is not sex with men per se, but rather forced sex that is the important risk factor for self-reported HIV infection among the participating women. HIV/AIDS policy should also address the needs of lesbian, bisexual and other women who have sex with women.

## Introduction

Because the risk of sexual transmission of HIV between women is assumed to be low if not nonexistent, lesbian women and other women who have sex with women (WSW) are generally not seen as vulnerable to HIV infection. Although a few case reports suggest the possibility of female to female transmission [1], [2], systematic evidence about the viability of this route of transmission is lacking [3]–[7]. The absence of such evidence has been attributed to a lack of attention for this mode of transmission, or the institutionalized exclusion of lesbian women from “risk group” categories [8], [9]. On many occasions and in several forums, lesbian activists have strongly objected to their omission and the de-prioritization of their risk for HIV transmission [10], [11]. This lack of attention might reinforce the myth of “lesbian immunity” [12]–[16], and the idea among lesbian women of not being at risk [13], [17], leaving them ill-equipped to protect themselves and their partners.

The accumulating evidence regarding the transmissible reproductive tract infections in lesbian women and other WSW warrants attention for HIV/AIDS among this population [18]. For instance, Singh and colleagues found an elevated prevalence of chlamydia trachomatis among WSW compared to women who reported sex with men only [19]. Studies furthermore have shown that human papillomavirus (HPV) is common among WSW, while evidence strongly suggests that HPV transmission occurs among WSW [20]–[22]. Finally, bacterial vaginosis (BV) in WSW was found to be associated with number of female sexual partners, but not with sex with men, suggesting that BV is sexually transmitted between women [23].

Despite the ongoing silence, the presence of HIV/AIDS in lesbian women and other WSW has been documented since the early 1990s, with prevalence rates that are sometimes higher than among their heterosexual counterparts [3], [15], [24]–[29]. This increased risk of HIV is usually associated with injection drug use and sexual involvement with men. For instance, Diaz et al. reported among female injection drug users a higher infection rate in WSW compared to exclusively heterosexual women; WSW were also more likely than heterosexual women to have had unprotected sex with an older male injection drug user [26]. That WSW more frequently engage in HIV risk practices is furthermore suggested by findings from a study in which WSW were compared with women who reported that they never had engaged in sexual interactions with other women. In this study WSW were more likely to report previous sexual contact with a homo−/bisexual man, or with an injection drug user [30]. Sexual contact with men seems to occur frequently among lesbian women and other WSW [31]–[33]: Diamant et al. reported, for instance, that in a sample of 6935 self-identified lesbians in the United States, over 77% had one or more lifetime male sexual partners, while close to 6% had a male sexual partner in the preceding year [34]. In the National Health and Nutrition Examination Surveys (NHANES), a series of cross-sectional surveys conducted by the National Center for Health Statistics to compile nationally representative statistics on the health of the US population, 9.6% and 84.4% of all women who identified as lesbian or homosexual had had sex with one or more men in the preceding year or during their life [31].

Most studies about lesbian women and other WSW and HIV/AIDS have focused on special populations, such as women who are HIV-positive and injection drug users (e.g. [35]–[37], and have been conducted in high income countries [37]. Studies of HIV/AIDS in general samples of WSW and WSW in middle and low income countries, some of which have the highest HIV prevalence worldwide, are rare [38]. In addition, HIV testing behavior in WSW has hardly been explored. To address this gap, we conducted a community-based survey among a sample of lesbian women and other WSW in Southern Africa to assess the prevalence of HIV-testing and HIV infection, and to identify the characteristics of women who reported to be HIV-positive among those that had been tested.

## Materials and Methods

### Ethics Statement

The study was approved by the Research Ethics Committee of the Human Sciences Research Council (South Africa) and the Internal Review Board of the New York State Psychiatric Institute (New York). A waiver of written consent for participation in this study was obtained (1) to protect participants’ confidentiality; (2) to increase the likelihood of participation; and (3) to ensure a diverse sample. Participation presented no more than minimal risk to participants while no identifying information was collected. Signed consent forms would themselves have presented a larger threat to the participant's privacy since they would be the only documentation linking the participant to a study about a potentially stigmatizing behavior (same-sex sexual behavior)? requiring signed consent forms also might have created a barrier to participation for some women who are less open about their sexuality. Study participation was explained and discussed with participants and each participant was given a study information sheet with contact information of the principal investigator as well as the South African Research Ethics Committee of the Human Sciences Research Council. According to approved procedures, oral consent was subsequently obtained and documented by having the recruiter sign a consent form without naming the individual. A few of the individual organizations involved in participant recruitment compensated participants for their time.

### Approach

The study was a collective endeavor of the participating community-based organizations [GALZ (Gays and Lesbians of Zimbabwe), LeGaBiBo (Lesbians, Gays and Bisexuals of Botswana), OutRight Namibia, Triangle Project, Durban Lesbian and Gay Community & Health Centre, Forum for the Empowerment of Women, OUT LGBT Well-Being and Behind the Mask, South Africa], a research team (Drs. Matebeni, Reddy, and Sandfort), and representatives of the Open Society Initiative for Southern Africa as the funding institution (Southey-Swartz and Tallis). Research questions, study design and assessment instruments were identified and developed collectively in two one-week meetings and regular conference calls. Fieldwork was conducted by the community-based organizations after staff was trained in recruitment of participants, conduct of the interview, and ethical procedures. Extensive communication by phone took place throughout the implementation of the study. The meaning of study findings and policy implications were explored in a three-day meeting following data collection.

### Study Participants

Eligibility criteria for study participation included being biologically female and 18 years or older, having had sex with a woman in the preceding year, and currently living in Botswana, Namibia, South Africa, or Zimbabwe. Women were recruited into the study by the community-based organizations that used announcements at relevant meetings and spaces, and (gay friendly) religious services to advertise the study. Potential participants were furthermore recruited by text messages, cellphone, email and Facebook, making use of organizational data bases. Additional participants were recruited through referral. Data collection took place between September and December, 2010.

### Assessment

Questionnaires were filled out individually or in small groups, in places that were experienced by the participants as ‘safe’; these places included offices of the community-based organizations, private homes of the participants themselves or their friends, university facilities, cars, or public spaces such as parks and restaurants. Questionnaire completion occurred in the presence of a fieldworker who first explained the purpose of the study and obtained consent.

In addition to questions about demographic characteristics, we assessed various aspects related to gender and sexuality. Sexual attraction was assessed with the question “Do you feel more sexually attracted to women or to men?” Women could choose from the following options: only to women, more to women than to men, to women and men equally, more to men than to women, and only to men. Because most women (70.5%) selected “Only to women” all other answers were combined in the category “Women and men” (this includes 2 women who chose “Only to men”). Regarding sexual orientation, women were asked: “In terms of your sexual orientation, what do you consider yourself?” Women could choose from the following alternatives: lesbian, bisexual, gay, heterosexual, and other. We combined “lesbian” (76.2%) with “gay” (0.7%) and categorized the remaining women as “other” (see File S2 for the complete questionnaire).

Given the fact that the expression of same-sex sexuality in the Southern African context quite often intersects with gender nonconformity, we also assessed women’s perception of themselves as masculine and feminine, with two three-item scales [39]. Women were asked to rate themselves on a 5-point scale (Not at all – Extremely) regarding how feminine they perceive themselves, how feminine they act, appear and come across to others, and how feminine their personality is. A parallel scale was used for the assessment of masculinity. Participants were offered the following definition: “Masculine refers to persons who feel, look and act like ‘real’ men or in a manner which most people think that men should be like. Feminine is the opposite of masculine and refers to what usually is expected from women. People, men or women, who look and behave like ‘real’ women are called feminine.” Cronbach’s alpha for both scales was.94 and.91, respectively.

In terms of the women’s sexual behavior, we assessed lifetime number of female partners (based on the women’s responses, answers were categorized as: 1, 2 to 4, 5 to 9, and 10 or more) and lifetime number of male partners (based on the responses, answers were categorized as: 0, 1, 2 or more). Experiences with transactional sex were assessed with the question “Sometimes people get something in return for having sex with other people. This can be a variety of things, including food, a place to sleep, money and a lot of other things. Have you ever had sex with a woman for any of the following reasons?” A parallel question was asked for transactional sex with men. Women who indicated having had sex at least once for any of these reasons were classified as having engaged in transactional sex with men only, with women only, and with both men and women. In addition to women’s sexual behavior, we assessed lifetime recreational drug use and use of needles for intravenous drugs.

Forced sexual experiences were assessed with the question “Has a man or boy ever made you have sex when you did not want to by using force or threatening to harm you or someone close to you? This man or boy could have been a stranger, someone you knew, but also your intimate partner.” A parallel question was asked for forced sex by women. Women who indicated having had at least once a forced sexual experience were classified as having forced sex by men only, by women only, and by both men and women.

### Data Analysis

We used logistic regression to test bivariate and multivariate associations of variables with testing status (never versus ever having been tested for HIV) and HIV serostatus (negative or positive; only among women with known serostatus). For the multivariate logistic regression analyses with ever having tested and HIV status as outcomes, variables were entered in the following blocs: demographic characteristics, psychosexual characteristics, drug use, number of sexual partners, transactional sex experiences, and experiences with forced sex. Only variables significantly associated with the outcome (p<.05) were included in a subsequent model. Outcomes of these analyses are available online (Tables A and B in File S1). Here we present outcomes of the multivariate logistic regression that included all variables that were bivariately associated with the outcome variables (p<.10); these outcomes are similar to the stepwise procedure. All analyses were conducted with SPSS version 18.

## Results

In total, 591 women who met the eligibility criteria participated in the study (see Table 1 for a description of the sample). Most women lived in South Africa. Although the women’s ages ranged from 18 to 65, the sample was relatively young. Almost half of the women had completed secondary education or less; the other women had been or were still involved in some kind of higher level education. Most women identified their race as black; for analytic purpose we combined white (5.9%) and Asian/Indian (2.9%) women into the category “other”). About a third of the women were full-time employed and another third was unemployed. Almost half of the women reported to have a regular income. More than a third of the women were covered by some kind of health insurance.

**Table 1:** Characteristics of Southern African WSW who have and who have not been tested for HIV.

	Total (%)			Not Tested	Tested (%)	Unadjusted		Adjusted	
	(n = 591)			(n = 128)	(n = 463)	OR (95% CI)	p-value	OR (95% CI)	p-value
Age (mean) (SE)	26.0 (.27)			24.5 (.63)	26.4 (.30)	1.05 (1.02–1.09)	.004	1.01 (0.97–1.06)	.531
Race									
Black	464 (78.8%)			97	367 (79.1)	Referent			
Coloured	73 (12.4%)			21	52 (71.2)	0.65 (0.38–1.14)	.134		
Other	52 (8.8%)			10	42 (80.8)	1.11 (0.54–2.29)	.778		
Education									
Low	269 (45.7%)			67	202 (75.1)	Referent			
High	319 (54.3%)			60	259 (81.2)	1.43 (0.97–2.12)	.074	1.18 (0.73–1.89)	.503
Employment									
Full-time employed	192 (32.5%)			29	163 (84.9)	Referent			
Part-time employed	73 (12.4%)			15	58 (79.5)	0.69 (0.34–1.37)	.289	0.85 (0.38–1.89)	.689
Student	96 (16.2%)			25	71 (74.0)	0.51 (0.28–0.92)	.027	0.87 (0.35–2.14)	.756
Unemployed	199 (33.7%)			54	145 (72.9)	0.48 (0.29–0.79)	.004	1.00 (0.42–2.37)	.994
Other	31 (5.2%)			5	26 (83.9)	0.93 (0.33–2.61)	.883	1.21 (0.40–3.69)	.740
Regular income									
No	302 (51.9%)			84	218 (72.2)	Referent			
Yes	280 (48.1%)			43	237 (84.6)	2.12 (1.41–3.20)	<.001	1.40 (0.69–2.81)	.352
Health insurance									
No	374 (63.9%)			93	281 (75.1)	Referent			
Yes	211 (36.1%)			35	176 (83.4)	1.66 (1.08–2.56)	.021	1.28 (0.76–2.15)	.363
Country									
South Africa	360 (60.9%)			84	276 (76.7)	Referent			
Botswana	51 (8.6%)			5	46 (90.2)	2.80 (1.08–7.27)	.035	2.49 (0.87–7.14)	.091
Namibia	112 (19.0%)			23	89 (79.5)	1.18 (0.70–1.98)	.537	1.25 (0.67–2.34)	.489
Zimbabwe	68 (11.5%)			16	52 (76.5)	0.99 (0.54–1.82)	.972	0.83 (0.41–1.70)	.617
(Ever) married									
No	543 (91.9%)			122	421 (77.5)	Referent			
Yes	48 (8.1%)			6	42 (87.5)	2.03 (0.84–4.89)	.115		
Having children									
No	451 (76.3%)			111	340 (75.4)	Referent			
Yes	140 (2.37%)			17	123 (87.9)	2.36 (1.36–4.10)	.002	1.72 (0.83–3.58)	.146
Sexual attraction									
Women only	416 (70.4%)			96	320 (76.9)	Referent			
Women and men	175 (29.6%)			32	143 (81.7)	1.34 (0.86–2.09)	.198		
Sexual identification									
Lesbian/gay	452 (76.9%)			101	351 (77.7)	Referent			
Other	136 (23.1%)			27	109 (80.1)	1.16 (0.72–1.87)	.537		
Gender orientation									
Perceived masculinity (mean) (SE)	2.57 (.05)			2.82 (.11)	2.50 (.06)	0.81 (0.69–0.95)	.011	0.90 (0.72–1.12)	.353
Perceived femininity (mean) (SE)	2.93 (.05)			2.62 (.12)	3.01 (.06)	1.28 (1.09–1.50)	.003	1.10 (0.89–1.36)	.377
Recreational drug use (lifetime)									
No	295 (49.9%)			70	225 (76.3)	Referent			
Yes	296 (50.1%)			58	238 (80.4)	1.28 (0.86–1.89)	.223		
Used needles for IV drugs (lifetime)									
No	576 (97.5%)			125	451 (78.3)	Referent			
Yes	15 (2.5%)			3	12 (80.0)	1.11 (0.31–3.99)	.875		
Number of female partners									
1	53 (9.0%)			18	35 (66.0)	Referent			
2 to 4	185 (31.3%)			36	149 (80.5)	2.13 (1.08–4.18)	.028	2.42 (1.12–5.26)	.025
5 to 9	178 (30.1%)			37	141 (79.2)	1.96 (1.00–3.85)	.050	3.19 (1.45–7.03)	.004
10 or more	175 (29.6%)			37	138 (78.9)	1.92 (0.98–3.77)	.058	3.94 (1.76–8.86)	.001
Number of male partners									
0	312 (52.8%)			93	219 (70.2)	Referent			
1	84 (14.2%)			12	72 (85.7)	2.55 (1.32–4.92)	.005	1.81 (0.85–3.85)	.126
2 or more	195 (33.0%)			23	172 (88.2)	3.18 (1.93–4.40)	<.001	2.27 (1.18–4.36)	.014
Transactional sex (lifetime)									
None	481 (81.4%)			98	383 (79.6)	Referent			
With men only	20 (3.4%)			3	17 (85.0)	1.45 (0.42–5.05)	.559	0.73 (0.18–2.87)	.648
With women only	48 (8.1%)			17	31 (64.6)	0.47 (0.25–0.88)	.018	0.45 (0.21–0.95)	.035
With both men and women		42 (7.1%)		10	32 (76.2)	0.82 (0.39–1.72)	.598	0.29 (0.11–0.78)	.014
Forced sex (lifetime)									
None	407 (68.9%)			92	315 (77.4)	Referent			
By men only	88 (14.9%)			12	76 (86.4)	1.85 (0.96–3.55)	.064	1.59 (0.73–3.12)	.266
By women only	39 (6.6%)			14	25 (64.1)	0.52 (0.26–1.04)	.066	0.83 (0.37–1.85)	.650
By both men and women	57 (9.6%)			10	47 (82.5)	1.37 (0.67–2.82)	.389	1.70 (0.67–4.34)	.267

OR, odds ratio; CI, confidence interval; SE, standard error.

Almost one in every 10 women reported to currently being married or to have been married in the past. About a quarter of the women reported to be the biological parent of children. While most women reported to be in an ongoing intimate relationship, a third was currently living with the intimate partner. The majority of women said they were only sexually attracted to women and identified as lesbian or gay. Almost half of the women reported to ever have had consensual sex with a man; almost 1 in 5 women had consensual sex with a man in the preceding year. Transactional sex with men was reported by 10.5% of the women and transactional sex with women was reported by 15.2% of the women; ‘money’ was the most frequently reported reason for transactional sex with men as well as with women. Forced sex by men (lifetime) was reported by a quarter of the women and lifetime forced sex by women was reported by one of every 6 women. The (lifetime) use of any recreational drug was reported by half of all women (the most frequently reported drug was marijuana; use of any other drugs was only reported by small groups of women); a very small proportion of women reported to have used a needle to inject drugs into their body for other than medical reasons.

Most of the women in the sample had ever been tested for HIV (78.3%). Women who reported to have ever been tested for HIV were older (see Table 1), less likely to be a student or unemployed compared to being full-time employed, more likely to have a regular income and have health insurance Compared to women from South Africa, women from Botswana were more likely to have been tested. Women who were a biological parent were also more likely to have been tested. In terms of gender and sexual orientation and actual sexual behavior, we found that women who perceived themselves as more masculine were less likely to have been tested, while women who perceived themselves as relatively more feminine were more likely to have been tested. Compared to women who only had one female sexual partner (lifetime), women with 2 to 4 partners and 5 to 9 were more likely to ever have been tested. Compared to women who never had any male sexual partners, women who had had one male sexual partner or 2 or more partners were more likely to have been tested. Finally, women who had engaged in transactional sex with women only (lifetime) were less likely to have been tested compared to women who never engaged in transactional sex.

Women who had never been tested for HIV were asked to indicate the main reason for not having been tested. As shown in Table 2, the two reasons endorsed most frequently seem to be in contrast with each other. A majority of untested women indicated that they had not been tested out fear of finding out the test results. At the same time, 39.0% of the untested women indicated that they think that they are not at risk. Other reasons were mentioned less frequently. Still, 12.2% of the untested women mentioned potential discrimination and 6.5% of the women mentioned potential embarassment as a reason for not having been tested. Practical barriers such as costs of the test and not knowing where to go were rarely mentioned.

**Table 2:** Reasons for not getting tested for HIV among untested women (n = 128).

Reason	%
Fear of finding out results	44.7
I don't think I am at risk	39.0
I fear being judged/dicriminated when asking for test	12.2
I always use protection	8.9
I am embarrassed that people will think I am lesbian or bisexual	6.5
Cost of having the test done	2.4
Don't know where to go	1.6

In the multivariate analysis, none of the demographic and sexual and gender orientation variables were significantly associated with ever having been tested for HIV (Table 2). The factors still associated with having been tested related to the women’s sexual behavior. Compared to women who only had one female sexual partner (lifetime), women with 2 to 4 and 5 to 9 partners were more likely to ever have been tested. Compared to women who never had any male sexual partners, women who had had 2 or more partners were more likely to have been tested. Women who reported ever engaging in transactional sex with women only or with both women and men were less likely to have been tested compared to women who never engaged in transactional sex.

Thirty-four (7.3%) of the 463 women who had been tested for HIV reported not to know their serostatus. Of the 429 women who knew their serostatus, 41 women (9.6%; 6.9% of the sample including the women who had never been tested for HIV or who did not know their serostatus) reported to be HIV-positive. Bivariately, women living with HIV/AIDS were more likely to be older, to be married or have been married, and to be a biological parent than women who tested negative (see Table 3). In terms of sexual orientation and gender identification, lesbian women living with HIV/AIDS were more likely to only be attracted to women (rather than to both women and men), and to see themselves comparatively as less feminine.

**Table 3:** Characteristics of Southern African WSW who tested negative or positive for HIV.

	Tested Negative	Tested Positive (%)	Unadjusted		Adjusted	
	(n = 388)	(n = 41)	OR (95% CI)	p-value	OR (95% CI)	p-value
Age (mean) (SE)	26.2 (.32)	29.8 (1.13)	1.07 (1.03–1.12)	.001	1.09 (1.01–1.16)	.019
Race						
Black	299	39 (11.5)	Referent		Referent	
Coloured	48	1 (2.0)	0.16 (0.02–1.19)	.073	0.13 (0.02–1.09)	.060
Other	39	1 (2.5)	0.20 (0.03–1.47)	.113	0.25 (0.03–2.49)	.236
Education						
Low	159	21 (11.7)	Referent			
High	228	19 (7.7)	0.63 (0.33–1.21)	.167		
Employment						
Full-time employed	140	15 (9.7)	Referent			
Part-time employed	48	5 (9.4)	0.97 (0.34–2.82)	.959		
Student	61	2 (3.2)	0.31 (0.07–1.38)	.123		
Unemployed	118	15 (11.3)	1.19 (0.56–2.53)	.658		
Other	21	4 (16.0)	1.78 (0.54–5.87)	.345		
Regular income						
No	173	23 (11.7)	Referent			
Yes	208	17 (7.6)	0.62 (0.32–1.19)	.148		
Health insurance						
No	230	30 (11.5)	Referent		Referent	
Yes	153	10 (6.1)	0.50 (0.24–1.06)	.069	0.81 (0.33–1.97)	.640
Country						
South Africa	228	28 (10.9)	Referent			
Botswana	46	0 (.0)	0.00 (0.00)	.997		
Namibia	65	10 (13.3)	1.25 (0.58–2.71)	.568		
Zimbabwe	49	3 (5.8)	0.50 (0.15–1.71)	.267		
(Ever) married						
No	355	33 (8.5)	Referent		Referent	
Yes	33	8 (19.5)	2.61 (1.11–6.11)	.027	2.39 (0.79–7.17)	.121
Having children						
No	288	22 (7.1)	Referent		Referent	
Yes	100	19 (16.0)	2.49 (1.29–4.79)	.006	1.03 (0.40–2.66)	.949
Sexual attraction						
Women only	256	34 (11.7)	Referent		Referent	
Women and men	132	7 (5.0)	0.40 (0.17–0.93)	.032	0.44 (0.16–1.20)	.110
Sexual identification						
Lesbian/gay	286	35 (10.9)	Referent			
Other	99	6 (5.7)	0.50 (0.20–1.21)	.124		
Gender identification						
Perceived masculinity (mean) (SE)	2.49 (.06)	2.56 (.23)	1.05 (0.79–1.38)	.744		
Perceived femininity(mean) (SE)	3.08 (.06)	2.60 (.24)	0.73 (0.56–0.96)	.026	0.79 (0.58–1.08)	.142
Recreational drug use (lifetime)						
No	185	24 (11.5)	Referent			
Yes	203	17 (7.7)	0.65 (0.34–1.24)	.189		
Used needles for IV drugs (lifetime)						
No	381	39 (9.3)	Referent			
Yes	7	2 (22.2)	2.79 (0.56–13.90)	.210		
Number of female partners						
1	28	4 (12.5)	Referent			
2 to 4	128	11 (7.9)	0.60 (0.18–2.03)	.412		
5 to 9	124	11 (8.1)	0.62 (0.18–2.09)	.442		
10 or more	108	15 (12.2)	0.97 (0.30–3.16)	.963		
Number of male partners						
0	178	17 (8.7)	Referent			
1	66	4 (5.7)	0.64 (0.21–1.96)	.428		
2 or more	144	20 (12.2)	1.45 (0.74–2.88)	.282		
Transactional sex (lifetime)						
None	327	28 (7.9)	Referent		Referent	
With men only	15	2 (11.8)	1.56 (0.34–7.16)	.569	0.61 (0.10–3.85)	.596
With women only	26	2 (7.1)	0.90 (0.20–3.98)	.888	0.43 (0.08–2.19)	.306
With both men and women	20	9 (31.0)	5.26 (2.19–12.62)	<.001	2.25 (0.69–7.29)	.178
Forced sex (lifetime)						
None	275	15 (5.2)	Referent		Referent	
By men only	61	10 (14.1)	3.01 (1.29–7.01)	.011	3.33 (1.28–8.70)	.014
By women only	20	5 (20.0)	4.58 (1.51–13.90)	.007	4.19 (1.21–14.47)	.024
By both men and women	32	11 (25.6)	6.30 (2.67–14.89)	<.001	5.48 (1.70–17.63)	.004

OR, odds ratio; CI, confidence interval; SE, standard error.

In terms of behavioral risk factors, neither the number of female partners, nor the number of male partners was associated with being HIV-positive. Compared to women who never engaged in transactional sex, women who during their lifetime had had transactional sex with both men and women were more likely to be HIV-positive. Compared to women who did not report any forced sexual experiences, women who had had forced sex only by men, only by women, or by both men and women, were more likely to be HIV-positive. Lifetime drug use, recreational or intravenous, did not increase the odds of being HIV-positive.

In the multivariate analysis, none of the demographic and sexual and gender orientation variables were anymore associated with HIV serostatus except for age: lesbian women living with HIV/AIDS were likely to be older. Having engaged in transactional sex was also no longer associated with HIV serostatus. The only factor still associated with living with HIV was ever having had forced sexual experiences. Compared to women who did not report any lifetime forced sexual experiences, women who had had forced sex only by men, only by women, or by both men and women, were more likely to report being HIV-positive. To check whether our findings were affected by the absence of any HIV positive women in Botswana, we ran all analyses excluding the women from Botswana. This did not seem to affect the outcomes: age and forced sex experiences only by men, only by women, and by both men and women were all associated with self-reported positive HIV status.

In trying to identify the most likely transmission route for the 41 women who were HIV-positive, we found that 2 of them reported to ever have used needles for intravenous drug use. Twenty-six women reported to ever have had consensual sex with and/or forced sex by men. Based on the available data we could not identify a transmission route for 13 of the infected women.

## Discussion

The majority of lesbian women and other women who have sex with women in this sample had tested for HIV at least once. Indicators of sexual risk were sometimes positively and at other times negatively related to having been tested. For instance, women were more likely to have been tested if they had had sex with men, but they were less likely to have been tested if they had engaged in transactional sex with men and women; some of the HIV testing could have taken place in the context of prenatal care. As in studies among South African MSM [40], not having tested for HIV also seemed an indication of social vulnerability: unemployed women, women without regular income and health insurance, and gender non-conforming women were less likely to have been tested. The fact that fear of finding out HIV test results was the most frequently endorsed reason among the women who had not been tested suggests that there is an awareness of the possibility that one might be infected. We were not able to assess the validity of the second reason that was most frequently endorsed: the perception of not being at risk.

About one in ten of the women who knew their HIV status reported to be living with HIV. From the perspective that lesbian women are invulnerable to HIV, this prevalence would seem high. At the same time, it is lower than the general HIV prevalence in each of the four countries [41]. The observed prevalence of 9.6% could be an underestimate, because the serostatus that women reported was not necessarily recent and because the HIV prevalence among the untested women could have been higher than among the tested women. Surprisingly, the only factor that was independently associated with a self-reported positive HIV status among the tested women was having had forced sex, not just by men but also by women. Because of the study’s cross-sectional design, it is not possible to determine whether the forced sex experiences preceded the infection or whether they were the actual cause of the infection. It is likely, that forced sex is associated with other factors that increase women’s risk. Various studies have shown that WSW not only have more health risk factors and lower levels of protective factors compared to non-WSW [32], [36], [42], but that health risk factors also tend to cluster. Lewis [43] for instance found how substance use, interpersonal violence, and discrimination tend to co-occur among women who have sex with women.

In terms of the source of infection, we could not identify the most likely transmission route of about a third of the women. We cannot rule out that these women were infected at birth or through medical procedures. It is, however, also possible that these women were infected in sexual interactions with other women.

The study findings should be seen in the context of some study limitations. First, the sample studied is a convenience sample; it is likely that women who were more comfortable with their same-sex sexuality and better connected to other women who have sex with women were more likely to participate. Based on the judgments of the community-based organizations, younger women, black women, and women living in urban areas were likely to be overrepresented. It is not clear how this affected the reported findings. Furthermore, all findings, including HIV status, are based on self-report. Although studies showed that self-reports of HIV test results are reliable [44]–[46], we cannot exclude the possibility that some women, for instance women with forced sex experiences, were more likely to think they were HIV positive than women without such experiences. Another limitation is that the questionnaire was only offered in an English version. It is likely that this restricted participation of women with little command of English and it might have caused some problems in filling out the questionnaires.

Our findings make clear that HIV affects Southern African lesbian, bisexual and other women who have sex with women. This should have several implications for HIV prevention and health care, and HIV/AIDS policies more generally. Most importantly, our data make clear, as other studies have done before [31]–[34], [47], that sexual identification as ‘lesbian’ does not exclude any sexual involvement with men, both on a lifetime basis as well as more recently. This implies that prevention messages about HIV risk should be made relevant for WSW in order for them to feel included and addressed. Furthermore, reporting of same-sex sexuality should not deter health care providers from addressing sexual risk [34], [48], while health care providers should be capacitated to provide non-judgmental care to WSW, who usually experience formidable barriers to access critical preventive health care and treatment services [49]. In general, in the Southern African context, HIV/AIDS policy should also address the needs of lesbian, bisexual and other women who have sex with women. This will be most effective if the heterogeneity of the population of WSW is recognized and programs are tailored in such a way that they best suit the needs of WSW locally.

Our findings furthermore suggest the need for research that addresses WSW’s awareness of HIV and STI risk and their engagement in sexual risk practices; few studies [12], [50] have done so thus far, while some studies have shown that adoption of safer sex practices among WSW is rare (e.g., [51]). In addition, we need to understand how accurate, appropriate and complete counseling could be provided to WSW living with HIV in order to prevent transmission to other female sex partners. Furthermore, our findings suggest a need to better understand forced sexual experiences of WSW with both men and women and their consequences for health behaviors and general health.

## Supporting Information

File S1 **Tables A and B.** Table A Characteristics of Southern African WSW who have and who have not been tested for HIV. Table B Characteristics of Southern African WSW who tested negative or positive for HIV.
(DOC)

File S2 **Study questionnaire.**
(PDF)
