# Supplementary material for: Forced Sexual Experiences as Risk Factor for Self-Reported HIV Infection among Southern African Lesbian and Bisexual Women
Source: PLoS One. 2013 Jan 9;8(1):e53552. doi: 10.1371/journal.pone.0053552 (PMC3541146; doi:10.1371/journal.pone.0053552)
Supplement: File S1 — Tables A and B. Table A Characteristics of Southern African WSW who have and who have not been tested for HIV. Table B Characteristics of Southern African WSW who tested negative or positive for HIV. (DOC) [file pone.0053552.s001.doc]

**Supplementary Table A Characteristics of Southern African WSW who have and who have not been tested for HIV**

|  | Model 1 | | | | | Model 2 | Model 3 | Model 4 | Model 5 | Model 6 |
| --- | --- | --- | --- | --- | --- | --- | --- | --- | --- | --- |
|  | AOR (95% CI) | | | | | AOR (95% CI) | AOR (95% CI) | AOR (95% CI) | AOR (95% CI) | AOR (95% CI) |
| Age (mean) (SE) | 1.04 (1.00-1.08)* | | | | | 1.04 (0.99-1.08) |  |  |  |  |
| Race |  | | | | |  |  |  |  |  |
| Black | Referent | | | | |  |  |  |  |  |
| Coloured | 0.58 (0.31-1.05) | | | | |  |  |  |  |  |
| Other | .74 (0.33-1.64) | | | | |  |  |  |  |  |
| Education |  | | | | |  |  |  |  |  |
| Low | Referent | | | | |  |  |  |  |  |
| High | 1.22 (0.78-1.90) | | | | |  |  |  |  |  |
| Employment |  | | | | |  |  |  |  |  |
| Full-time employed | Referent | | | | |  |  |  |  |  |
| Part-time employed | 0.91 (0.43-1.90) | | | | |  |  |  |  |  |
| Student | 0.96 (0.41-2.22) | | | | |  |  |  |  |  |
| Unemployed | 0.96 (0.44-2.10) | | | | |  |  |  |  |  |
| Other | 1.19 (0.41-3.50) | | | | |  |  |  |  |  |
| Regular income |  | | | | |  |  |  |  |  |
| No | Referent | | | | |  |  |  |  |  |
| Yes | 2.65 (0.86-3.20) | | | | |  |  |  |  |  |
| Health insurance |  | | | | |  |  |  |  |  |
| No | Referent | | | | |  |  |  |  |  |
| Yes | 1.37 (0.83-2.28) | | | | |  |  |  |  |  |
| Country |  | | | | |  |  |  |  |  |
| South Africa | Referent | | | | |  |  |  |  |  |
| Botswana | 2.27 (0.84-6.14) | | | | |  |  |  |  |  |
| Namibia | 1.37 (0.77-2.41) | | | | |  |  |  |  |  |
| Zimbabwe | 0.99 (0.51-1.90) | | | | |  |  |  |  |  |
| (Ever) married |  | | | | |  |  |  |  |  |
| No |  | | | | | Referent |  |  |  |  |
| Yes |  | | | | | 1.50 (0.55-4.12) |  |  |  |  |
| Having children |  | | | | |  |  |  |  |  |
| No |  | | | | | Referent |  |  |  |  |
| Yes |  | | | | | 1.66 (0.91-3.02) |  |  |  |  |
| Sexual attraction |  | | | | |  |  |  |  |  |
| Women only |  | | | | | Referent |  |  |  |  |
| Women and men |  | | | | | 1.00 (0.50-2.01) |  |  |  |  |
| Sexual identification |  | | | | |  |  |  |  |  |
| Lesbian/gay |  | | | | | Referent |  |  |  |  |
| Other |  | | | | | 0.75 (0.36-1.57) |  |  |  |  |
| Gender orientation |  | | | | |  |  |  |  |  |
| Perceived masculinity (mean) (SE) |  | | | | | 0.91 (0.74-1.12) |  |  |  |  |
| Perceived femininity (mean) (SE) |  | | | | | 1.22 (0.99-1.49) |  |  |  |  |
| Recreational drug use (lifetime) | | |  | | |  |  |  |  |  |
| No |  | | | | |  | Referent |  |  |  |
| Yes |  | | | | |  | 1.28 (0.86-1.89) |  |  |  |
| Used needles for IV drugs (lifetime) | | | | |  |  |  |  |  |  |
| No |  | | | | |  | Referent |  |  |  |
| Yes |  | | | | |  | 1.05 (0.29-3.78) |  |  |  |
| Number of female partners |  | | | | |  |  |  |  |  |
| 1 |  | | | | |  |  | Referent | Referent | Referent |
| 2 to 4 |  | | | | |  |  | 2.25 (1.11-4.53)* | 1.99 (0.98-4.08) | 2.11 (1.02-4.35)* |
| 5 to 9 |  | | | | |  |  | 2.50 (1.23-5.07)* | 2.45 (1.20-5.01)* | 2.59 (1.25-5.35)** |
| 10 or more |  | | | | |  |  | 2.64 (1.29-5.37)** | 2.81 (1.36-5.80)** | 3.06 (1.47-6.38)** |
| Number of male partners |  | | | | |  |  |  |  |  |
| 0 |  | | | | |  |  | Referent | Referent | Referent |
| 1 |  | | | | |  |  | 2.69 (1.38-5.26)** | 2.87 (1.45-5.66)** | 2.63 (1.32-5.20)** |
| 2 or more |  | | | | |  |  | 3.50 (2.10-5.85)*** | 3.88 (2.24-6.72)*** | 3.59 (2.05-6.30)*** |
| Transactional sex (lifetime) |  | | | | |  |  |  |  |  |
| None |  | | | | |  |  |  | Referent | Referent |
| With men only |  | | | | |  |  |  | 0.76 (0.20-2.85) | 0.73 (0.19-2.76) |
| With women only |  | | | | |  |  |  | 0.47 (0.24-0.92)* | 0.47 (0.24-0.95)* |
| With both men and women | | | |  | |  |  |  | 0.47 (0.21-1.08) | 0.29 (0.15-0.94)* |
| Forced sex (lifetime) |  | | | | |  |  |  |  |  |
| None |  | | | | |  |  |  |  | Referent |
| By men only |  | | | | |  |  |  |  | 1.36 (0.69-2.70) |
| By women only |  | | | | |  |  |  |  | 0.66 (0.32-1.40) |
| By both men and women | |  | | | |  |  |  |  | 1.70 (0.71-4.02) |

AOR, adjusted odds ratio; CI, confidence interval; SE, standard error.

** p* < .05; *** p* < .01; **** p* < .001

**Supplementary Table B Characteristics of Southern African WSW who tested negative or positive for HIV**

|  | Model 1 | | | | | | Model 2 | Model 3 | Model 4 | Model 5 | Model 6 |
| --- | --- | --- | --- | --- | --- | --- | --- | --- | --- | --- | --- |
|  | AOR (95% CI) | | | | | | AOR (95% CI) | AOR (95% CI) | AOR (95% CI) | AOR (95% CI) | AOR (95% CI) |
| Age (mean) (SE) | 1.13 (1.07-1.20)*** | | | | | | 1.08 (1.01-1.16)* | 1.10 (1.04-1.15)*** | 1.10 (1.04-1.16)*** | 1.09 (1.03-1.15)*** | 1.11 (1.05-1.17)*** |
| Race |  | | | | | |  |  |  |  |  |
| Black | Referent | | | | | | Referent | Referent | Referent | Referent | Referent |
| Coloured | 0.08 (0.01-0.67)* | | | | | | 0.10 (0.01-0.82)* | 0.09 (0.01-0.71)* | 0.90 (0.01-0.72)* | 0.10 (0.01-0.76)* | 0.10 (0.01-0.81)* |
| Other | 0.18 (0.21-1.57) | | | | | | 0.16 (0.02-1.51) | 0.10 (0.01-0.78)* | 0.10 (0.01-0.80)* | 0.11 (0.01-0.88)* | 0.14 (0.02-1.16) |
| Education |  | | | | | |  |  |  |  |  |
| Low | Referent | | | | | |  |  |  |  |  |
| High | 0.71 (0.32-1.61) | | | | | |  |  |  |  |  |
| Employment |  | | | | | |  |  |  |  |  |
| Full-time employed | Referent | | | | | |  |  |  |  |  |
| Part-time employed | 1.22 (0.35-4.31) | | | | | |  |  |  |  |  |
| Student | 0.29 (0.05-1.80) | | | | | |  |  |  |  |  |
| Unemployed | 0.96 (0.14 -1.74) | | | | | |  |  |  |  |  |
| Other | 2.02 (0.49-8.39) | | | | | |  |  |  |  |  |
| Regular income |  | | | | | |  |  |  |  |  |
| No | Referent | | | | | | Referent |  |  |  |  |
| Yes | 0.19 (0.06-0.58)** | | | | | | 0.52 (0.23-1.15) |  |  |  |  |
| Health insurance |  | | | | | |  |  |  |  |  |
| No | Referent | | | | | |  |  |  |  |  |
| Yes | 0.92 (0.37-2.32) | | | | | |  |  |  |  |  |
| Country |  | | | | | |  |  |  |  |  |
| South Africa | Referent | | | | | | Referent |  |  |  |  |
| Botswana | 0.00 (0.00-0.00) | | | | | | 0.00 (0.00-0.00) |  |  |  |  |
| Namibia | 0.87 (0.34-2.25) | | | | | | 1.14 (0.46-2.87) |  |  |  |  |
| Zimbabwe | 0.24 (0.06-0.97)* | | | | | | 0.13 (0.02-1.09) |  |  |  |  |
| (Ever) married |  | | | | | |  |  |  |  |  |
| No |  | | | | | | Referent | Referent | Referent | Referent |  |
| Yes |  | | | | | | 3.25 (1.05-10.04)* | 2.04 (0.77-5.43) | 2.00 (0.74-5.40) | 2.13 (0.79-5.73) |  |
| Having children |  | | | | | |  |  |  |  |  |
| No |  | | | | | | Referent |  |  |  |  |
| Yes |  | | | | | | 1.75 (0.73-4.22) |  |  |  |  |
| Sexual attraction |  | | | | | |  |  |  |  |  |
| Women only |  | | | | | | Referent |  |  |  |  |
| Women and men |  | | | | | | 0.44 (0.11-1.76) |  |  |  |  |
| Sexual identification |  | | | | | |  |  |  |  |  |
| Lesbian/gay |  | | | | | | Referent |  |  |  |  |
| Other |  | | | | | | 1.13 (0.26-4.81) |  |  |  |  |
| Gender orientation |  | | | | | |  |  |  |  |  |
| Perceived masculinity (mean) (SE) |  | | | | | | 0.82 (0.56-1.19) |  |  |  |  |
| Perceived femininity (mean) (SE) |  | | | | | | 0.77 (0.55-1.10) |  |  |  |  |
| Recreational drug use (lifetime) | | | | |  | |  |  |  |  |  |
| No |  | | | | | |  | Referent |  |  |  |
| Yes |  | | | | | |  | 0.74 (0.37-1.46) |  |  |  |
| Used needles for IV drugs (lifetime) | | | | | |  |  |  |  |  |  |
| No | |  | | | | |  | Referent |  |  |  |
| Yes | |  | | | | |  | 2.84 (0.53-15.25) |  |  |  |
| Number of female partners | | |  | | | |  |  |  |  |  |
| 1 | |  | | | | |  |  | Referent |  |  |
| 2 to 4 | |  | | | | |  |  | 0.77 (0.20-2.92) |  |  |
| 5 to 9 | |  | | | | |  |  | 0.76 (0.20-2.90) |  |  |
| 10 or more | |  | | | | |  |  | 1.04 (0.28-3.86) |  |  |
| Number of male partners | |  | | | | |  |  |  |  |  |
| 0 | |  | | | | |  |  | Referent |  |  |
| 1 | |  | | | | |  |  | 2.69 (1.38-5.26) |  |  |
| 2 or more | |  | | | | |  |  | 3.50 (2.10-5.85) |  |  |
| Transactional sex (lifetime) | | |  | | | |  |  |  |  |  |
| None | |  | | | | |  |  |  | Referent | Referent |
| With men only | |  | | | | |  |  |  | 1.03 (0.20-5.22) | 0.78 (0.15-4.03) |
| With women only | |  | | | | |  |  |  | 0.83 (0.18-3.80) | 0.41 (0.08-2.02) |
| With both men and women | | | | | |  |  |  |  | 3.96 (1.56-10.04)** | 1.92 (0.64-5.74) |
| Forced sex (lifetime) | | | |  | | |  |  |  |  |  |
| None | | | |  | | |  |  |  |  | Referent |
| By men only | | | |  | | |  |  |  |  | 1.36 (0.69-2.70)* |
| By women only | | | |  | | |  |  |  |  | 0.66 (0.32-1.40)* |
| By both men and women | | | |  | | |  |  |  |  | 1.70 (0.71-4.02)** |

AOR, adjusted odds ratio; CI, confidence interval; SE, standard error.

** p* < .05; *** p* < .01; **** p* < .001
