# Supplementary material for: Forced Sexual Experiences as Risk Factor for Self-Reported HIV Infection among Southern African Lesbian and Bisexual Women
Source: PLoS One. 2013 Jan 9;8(1):e53552. doi: 10.1371/journal.pone.0053552 (PMC3541146; doi:10.1371/journal.pone.0053552)
Supplement: File S2 — Study questionnaire. (PDF) [file pone.0053552.s002.pdf]

RAISING THE VISIBILITY OF LESBIAN, BISEXUAL AND OTHER WOMEN WHO HAVE SEX WITH WOMEN

Thank you for your interest and participating in our study. We want you to know that your answers to the questions in this questionnaire are important! They will help all of us to think about the best way lesbian, bisexual, and other women who have sex with women can be supported in avoiding health problems and living happy lives. Your responses will help to create better services for women.

There are a few things that you have to know before you begin filling in the questionnaire. Your participation is voluntary, so it is up to you whether you want to answer the questions in this list. You help us the most, though, if you try to answer all the questions that apply to you (we make it clear when questions can be skipped).
We also want you to try to answer the questions as honestly as possible. Remember, your answers are strictly confidential; we do not ask for your name or any other information that could link the questionnaire to you.

There are different kinds of questions in this questionnaire. For most questions you will have to tick the one answer that applies best to you by putting an X in the box before the answer. For instance, in response to the question about your race/population group, you put an X in the box before "Black" if you belong to that group:

1 How would you describe yourself in terms of race/population group?

- 1☒ Black
- 2☐ Coloured
- 3☐ Indian
- 4☐ White
- 5☐ Other; please specify _______________

For other questions you will have to put an X in the box in a specific column. For instance, you will be asked how often in the past 4 weeks you felt tired for no good reason. If you felt that way "Some of the time," you put an X in the box in the column "Some of the time." See the example below:

	None of the time	A little of the time	Some of the time	Most of the time	All of the time
a) In the past 4 weeks, about how often did you feel tired for no good reason?	1☐	2☐	3☒	4☐	5☐

In almost all cases you are only expected to give one answer. In a few cases you are asked to tick as many answers that apply to you. That is only the case if you are specifically asked to do so.

For a few questions you will have to fill in your answer (e.g., "How old are you?"). Sometimes, when you tick a box, you will be asked to specify your answer; you can then write your answer on the dotted line.

We hope that answering the questions in this list will be of interest to you. Thank you again for your participation!

Z	A			

ABOUT YOU

1 How old were you at your last birthday?

___ ___ years

2 What is your nationality?

1☐ South African
2☐ Motswana
3☐ Namibian
4☐ Zimbabwean
5☐ Other; please specify _______________

3 In which country do you currently live?

1☐ South Africa
2☐ Botswana
3☐ Namibia
4☐ Zimbabwe
5☐ Other; please specify _______________

4 How would you describe yourself in terms of your race?

1☐ Black/African
2☐ Coloured
3☐ White
4☐ Asian/Indian

5 What is the main language spoken in your home?

1☐ English
2☐ Afrikaans
3☐ IsiXhosa
4☐ IsiZulu
5☐ Sesotho
6☐ Setswana
7☐ Sepedi
8☐ SiSwati
9☐ Tshivenda
10☐ Xitsonga
11☐ IsiNdebele
12☐ Other; please specify _______________

6 What is your religion? (tick one or two)

1☐ African traditional
2☐ Muslim
3☐ Christian
4☐ Spiritual
5☐ None
6☐ Other; please specify _______________

7 Do you consider yourself to have a physical disability (hearing, visual, mobility, etc.)?

1☐ Yes
2☐ No
3☐ Do not wish to say

8 What is the highest level of school you attended: primary, secondary, or higher?

1☐ Primary
2☐ Secondary
3☐ Higher (university or other post matric inst.)

9 What is the highest (grade/year/qualification) you completed at that level?

Grade/year/qualification ________

10 What is your work status?

1☐ Full time employed
2☐ Part time employed
3☐ Student
4☐ Unemployed
5☐ Other; please specify _______________

11 Do you have regular income from work that you are doing (**excluding** pocket money, grants, etc.)?

- 1☐ Yes
- 2☐ No

12 **In the past 6 months**, how many hours per week did you work on average for wages?

- 1☐ None
- 2☐ 1-10 hours per week
- 3☐ 11-20 hours per week
- 4☐ 21-30 hours per week
- 5☐ 31 hours or more per week

13 What is the main source of your financial support, including food, rent, and other expenses?

- 1☐ My own job or salary
- 2☐ Someone else's job or salary
- 3☐ Grants or pensions
- 4☐ Other; please specify _______________

14 Do you currently have a regular sexual partner (such as a lover, spouse, or domestic partner)?

- 1☐ Yes, I have one regular sexual partner
- 2☐ Yes, I have two or more regular partners
- 3☐ No, but I have one or more casual sexual partners
- 4☐ No, I am not currently sexually active

15 **IF YOU ARE IN A RELATIONSHIP:** What is the sex of your partner?

- 1☐ Female
- 2☐ Male
- 3☐ Transgender

16 **IF YOU ARE IN A RELATIONSHIP:** Are you now living with your partner?

- 1☐ Yes
- 2☐ No

17 What is your current legal marital status?

- 1☐ Never married
- 2☐ Legally married → What is the sex of your partner: 1☐ Female 2☐ Male 3☐ Transgender
- 3☐ Legally separated
- 4☐ Divorced
- 5☐ Widowed
- 6☐ Do not wish to provide

18 How many children have you given birth to?

___ ___ children

19 Are you covered by any medical aid?

- 1☐ Yes
- 2☐ No

HOW YOU SEE YOURSELF AS A SEXUAL PERSON

20 Do you feel more sexually attracted to women or to men?

- 1☐ Only to women
- 2☐ More to women than to men
- 3☐ To women and men equally
- 4☐ More to men than to women
- 5☐ Only to men

21 Are your recent sexual experiences more with women or with men?

- 1☐ Only with women
- 2☐ More with women than with men
- 3☐ With women and men equally
- 4☐ More with men than with women
- 5☐ Only with men
- 6☐ I am currently not sexually active

22 In terms of your sexual orientation, what do you consider yourself?

- 1☐ Lesbian
- 2☐ Bisexual
- 3☐ Gay
- 4☐ Heterosexual
- 5☐ Other; please specify _______________

23 Were you born male or female?

- 1☐ Male
- 2☐ Female
- 3☐ Intersex (persons born with unclear or both male and female sex organs)

WHAT YOU KNOW ABOUT HIV/STI

24 As you know, there are diseases that can be transmitted through sexual contact. These diseases are called sexually transmitted diseases or infections (STI). Indicate below whether you think these diseases can be transmitted via the following routes. Tick the box of all that you think apply.

Women who have sex with each other can get a sexually transmitted disease through:

- 1☐ Skin-to-skin contact
- 2☐ Contact with vaginal fluids
- 3☐ Contact with menstrual blood

25 As you know, there is a disease called AIDS that is caused by a virus called HIV. The following statements are about the transmission of this virus. Indicate for each statement whether it is true or false.

	True	False	Don't know	
a)	1☐	2☐	3☐	Women can protect themselves from the virus that causes AIDS by using a condom correctly every time they have sexual intercourse with a man.
b)	1☐	2☐	3☐	Women who have sex with each other are not at risk for HIV transmission.
c)	1☐	2☐	3☐	A person can get HIV from mosquito bites.
d)	1☐	2☐	3☐	People can protect themselves from HIV by abstaining from sexual intercourse.
e)	1☐	2☐	3☐	People can get HIV by sharing a meal with someone who is infected.
f)	1☐	2☐	3☐	A person can get HIV by getting injections with a needle that was already used by someone else.
g)	1☐	2☐	3☐	People can avoid transmission of HIV by having anal sex.
h)	1☐	2☐	3☐	A pregnant woman infected with HIV or AIDS can transmit the virus to her unborn child.
i)	1☐	2☐	3☐	Women who have sex with each other can transmit HIV if they use sex toys (for example, vibrators) that are not cleaned.

YOUR SEXUAL EXPERIENCES WITH WOMEN

In this section, when we mention "sex," it refers to a variety of experiences that people have with each other: they range from stimulating a partner's vagina with the hand to sexual intercourse. To avoid misunderstanding we give as much as possible physical description of what we mean. We also refer to oral, vaginal and anal sex. Oral sex is caressing someone's private parts with the mouth. Vaginal sex is when a penis goes in someone's vagina. Anal sex refers to a penis going into someone's anus.

When you answer the following questions, remember that you can have different kinds of sexual partners: lovers, one-time partners, partners who you are married to, and partners that you just meet to have sex with. All these different kinds of partners are relevant!

26 In your **entire life**, how many women have you had sex of any kind with?

___ ___ women

27 Have you had sex with a woman in the past year (12 months)?

- 1☐ Yes
- 2☐ No → **IF "NO" CONTINUE WITH QUESTION 41..**

28 In the **past 3 months**, how many women have you had sex with (including a steady partner if applicable)?

___ ___ women → **IF "ZERO" CONTINUE WITH QUESTION 41.**

29 In the **past 3 months**, have you had sex with a woman that is your steady partner?

- 1☐ Yes
- 2☐ No

30 Listed below are several sexual activities. Please indicate for each activity whether you have engaged in this activity with a woman **in the past 3 months**.

	Yes	No	
a)	1☐	2☐	Your partner stimulated your vagina with her hand
b)	1☐	2☐	You stimulated your partner's vagina with your hand
c)	1☐	2☐	Your partner put her mouth or tongue on your vagina
d)	1☐	2☐	Your partner rubbed her vagina against your body
e)	1☐	2☐	You rubbed your vagina against your partner's body
f)	1☐	2☐	Your partner rubbed her vagina against your breast(s)
g)	1☐	2☐	You rubbed your vagina against your partner's breast(s)
h)	1☐	2☐	You put your mouth or tongue on your partner's vagina
i)	1☐	2☐	Your partner put her mouth or tongue on your anus/butt
j)	1☐	2☐	You put your mouth or tongue on your partner's anus/butt
k)	1☐	2☐	Your partner put her finger(s) in your vagina
l)	1☐	2☐	You put your finger(s) in your partner's vagina
m)	1☐	2☐	Your partner put her fist in your vagina
n)	1☐	2☐	You put your fist in your partner's vagina
o)	1☐	2☐	Your partner put her fist in your anus/butt
p)	1☐	2☐	You put your fist in your partner's anus/butt

31 During the **past 3 months**, have you and your partner used a dildo, vibrator, or other objects (sex toys) that were inserted into your vagina?

- 1☐ Yes
- 2☐ No

32 During the **past 3 months**, have you and your partner used a dildo, vibrator, or other objects (sex toys) that were inserted into your anus?

- 1☐ Yes
- 2☐ No

33 If you have used any dildos, vibrators, or sex toys in the **past 3 months**, did you clean them before they got (re)used?

- 1☐ Never
- 2☐ A few times
- 3☐ Often
- 4☐ Most of the times
- 5☐ Always

34 In the **past 3 months**, have you had sex with a woman while you were on your period (menstruation)?

- 1☐ Yes
- 2☐ No

35 In the **past 3 months**, have you had sex with a woman while she was on her period?

- 1☐ Yes
- 2☐ No

36 In the **past 3 months**, have you had any sex with a **woman who** was under the influence of alcohol or drugs?

- 1☐ Yes
- 2☐ No

37 In the **past 3 months**, have you had any sex with a woman while **you** were under the influence of alcohol or drugs?

- 1☐ Yes
- 2☐ No

HIV PREVENTION

38 In the **last 3 months**, did you do anything while having sex with other women to prevent possible transmission of HIV?

- 1☐ No, did not do anything
- 2☐ Yes

39 If you did something to prevent possible transmission of HIV, which of the following protective measures did you use? Tick all that apply.

- 1☐ Dental dams
- 2☐ Gloves
- 3☐ Cling wrap
- 4☐ Finger cots
- 5☐ Condoms for sex toys
- 6☐ Other; please specify _______________
- 7☐ Never used any protective measure

40 There are several reasons why women do not use protection when they have sex with each other. Please indicate below which reasons applied to you **in the past 3 months**. Tick all that apply.

- 1☐ It is not necessary to use protection
- 2☐ They were not available at the time
- 3☐ These things are difficult to get (too expensive, not available)
- 4☐ These things spoil the spontaneity, kill the flow
- 5☐ These things are unpleasant (smell, taste, feeling)
- 6☐ I did not use protection for other reasons

SEXUAL EXPERIENCES WITH MEN

41 The following questions are about sexual experiences with men. Here we are interested in experiences in which you participated freely. Sex can, again, mean a range of physical activities, including masturbation, oral, vaginal, and anal sex.

Have you ever in your life had any vaginal sex, oral sex, or anal sex with a man that you participated in freely?

- 1☐ Yes
- 2☐ No → **IF YOU NEVER HAD SEX WITH A MAN GO TO QUESTION 55**.

42 In your **entire life**, how many men have you had sex with?

___ ___ men

43 Have you had sex with a man **in the past year** (12 months)?

- 1☐ Yes
- 2☐ No → **IF "NO" CONTINUE WITH QUESTION 55.**

44 In the **past 3 months**, have you freely participated in any vaginal sex, oral sex, or anal sex with a man?

- 1☐ Yes
- 2☐ No

45 In the **past 3 months**, how many men have you had vaginal, oral, or anal sex with?

___ ___ men

46 In the **past 3 months**, have you had sex with a man or men because you wanted to get pregnant?

- 1☐ Yes
- 2☐ No

47 In the **past 3 months**, have you had vaginal sex with a man or men **without** using a condom?

- 1☐ Yes
- 2☐ No

48 In the **past 3 months**, have you had vaginal sex with a man or men in which condoms **were** used?

- 1☐ Yes
- 2☐ No

49 In the **past 3 months**, have you had anal sex with a man or men?

1☐ Yes
2☐ No

50 In the **past 3 months**, have you had any anal sex with a man or men in which a condom was used?

1☐ Yes
2☐ No

51 In the **past 3 months**, have you had any anal sex with a man or men **without** using a condom?

1☐ Yes
2☐ No

52 In the **past 3 months**, have you done anything to prevent pregnancy?

1☐ No
2☐ Yes, used condoms
3☐ Yes, I am on the pill
4☐ Yes, used other measure; please specify _______________

53 **In the past 3 months**, have you had any type of sex with a **man who** was under the influence of alcohol or drugs?

1☐ No
2☐ Yes

54 **In the past 3 months**, have you had any type of sex with a man while **you** were under the influence of alcohol or drugs?

1☐ No
2☐ Yes

YOUR IDEA OF RISK

55 If you think about your sexual experiences **in the past three months**, what do you think are the chances that you might have become infected with HIV?

1☐ No chance at all
2☐ A minor chance
3☐ A reasonable chance
4☐ A major chance
5☐ I have not had sex in the past three months

DRY SEX WITH WOMEN AND/OR MEN

“Dry sex” is sexual intercourse in which the woman dries her vagina using special powders, herbs, or douches in order to increase friction. The following 2 questions apply to sexual contact with women as well as with men.

56 Have you ever put herbs or other substances into your vagina before sex in order to dry, contract, or heat your vagina?

1☐ No → **IF YOU NEVER HAVE DONE THIS GO TO 59.**
2☐ Yes

57 Have you done this before having sex with a man or with a woman?

1☐ With a man
2☐ With a woman
3☐ With both a man and a woman

58 Have you ever experienced any of the following side effects after putting herbs/substances into your vagina before sex?

	**Yes**	**No**	
a)	1☐	2☐	Pain
b)	1☐	2☐	Unpleasant feeling in the vagina
c)	1☐	2☐	Tears or cuts in the vagina
d)	1☐	2☐	Excessive vaginal secretions
e)	1☐	2☐	Reduction of your own sexual arousal

SEX FOR MONEY AND OTHER THINGS

59 Sometimes people get something in return for having sex with other people. This can be a variety of things, including food, a place to sleep, money and a lot of other things.

Have you **ever** had sex of any kind **with a woman** for any of the following reasons? Please tick all that apply.

1☐ Food, clothes and/or cosmetics
2☐ A place to sleep
3☐ Drugs
4☐ Money
5☐ Other things; please specify _______________
6☐ Never had sex with a woman for these reasons

60 In the **past year**, have you had sex **with a woman** in return for food, clothes, cosmetics, a place to sleep, drugs, money, etc?

1☐ Yes
2☐ No

61 Have you **ever** had sex of any kind **with a man** for any of the following reasons? Please tick all that apply.

1☐ Food, clothes and/or cosmetics
2☐ A place to sleep
3☐ Drugs
4☐ Money
5☐ Other things; please specify _______________
6☐ Never had sex with a man for these reasons

62 In the **past year**, have you had sex **with a man** in return for food, clothes, cosmetics, a place to sleep, drugs, money, etc?

1☐ Yes
2☐ No

UNWANTED SEXUAL EXPERIENCES WITH MEN

63 Has a man or boy ever made you have sex when you did not want to by using force or threatening to harm you or someone close to you? This man or boy could have been a stranger, someone you knew, but also your intimate partner.

1☐ Yes
2☐ No → **IF YOU NEVER HAVE HAD SUCH EXPERIENCES GO TO QUESTION 71.**

64 How often would you say you have had such experiences with men or boys?

1☐ Only once
2☐ A few times
3☐ Regularly
4☐ Often
5☐ Very often

65 Do you think that these experiences ever happened because you are lesbian, bisexual, or butch?

1☐ Yes, always
2☐ Yes, sometimes
3☐ No, never

66 If you **ever** had any of the above negative experiences with men or boys, were these men or boys you knew before this happened or were these men or boys strangers to you?

1☐ I knew the man/boy
2☐ I did not know the man/boy
3☐ There were men/boys that I knew and that I didn't know

67 Have you ever had such negative experiences with a man who was (or is) your partner?

1☐ Yes
2☐ No

68 Looking back, how serious would you say these negative experiences with men or boys were?

- 1☐ Not serious at all
- 2☐ Somewhat serious
- 3☐ Serious
- 4☐ Very serious

69 Could you indicate what kind of negative sexual experiences you have had with men and/or boys?

	Yes	No	
a)	1☐	2☐	He/they put fingers or objects in my vagina
b)	1☐	2☐	He/they put fingers or objects in my anus
c)	1☐	2☐	He/they performed oral sex on me
d)	1☐	2☐	He/they made me perform oral sex on him/them
e)	1☐	2☐	He/they put his/their penis in my vagina
f)	1☐	2☐	He/they put his/their penis in my anus

70 Have you had any such negative experiences with a man or boy **in the past year** (12 months)?

- 1☐ Yes
- 2☐ No

UNWANTED SEXUAL EXPERIENCES WITH WOMEN

71 Has a woman or girl ever made you have sex when you did not want to by using force or threatening to harm you or someone close to you? Again, this woman or girl could have been a stranger, someone you knew, but also your intimate partner.

- 1☐ Yes
- 2☐ No → **IF YOU NEVER HAVE HAD SUCH EXPERIENCES GO TO QUESTION 78.**

72 How often would you say you have had such experiences with women or girls?

- 1☐ Only once
- 2☐ A few times
- 3☐ Regularly
- 4☐ Often
- 5☐ Very often

73 If you **ever** had any of the above negative experiences with women or girls, were these women or girls you knew before this happened or were these women or girls strangers to you?

- 1☐ I knew the woman/girl
- 2☐ I did not know the woman/girl
- 3☐ There were women/girls that I knew and that I didn't know

74 Have you ever had such negative experiences with a woman who was (or is) your partner?

- 1☐ Yes
- 2☐ No

75 Looking back, how serious would you say these negative experiences with women or girls were?

- 1☐ Not serious at all
- 2☐ Somewhat serious
- 3☐ Serious
- 4☐ Very serious

76 Could you indicate what kind of negative sexual experiences you have had with women and/or girls?

	Yes	No	
a)	1☐	2☐	She/they stimulated my vagina with her/their fingers
b)	1☐	2☐	She/they made me stimulate her/their vagina with my fingers
c)	1☐	2☐	She/they put fingers or objects in my vagina
d)	1☐	2☐	She/they put fingers or objects in my anus
e)	1☐	2☐	She/they made me perform oral sex on her/them
f)	1☐	2☐	She/they performed oral sex on me

77 Have you had any such negative experiences with a woman or girl **in the past year** (12 months)?

1☐ Yes
2☐ No

ALCOHOL USE

78 How often do you drink alcoholic beverages?

1☐ Never
2☐ Once per month or less
3☐ Two to four times a month
4☐ Two to three times a week
5☐ Four or more times a week

79 How often do you have six or more alcoholic drinks on one occasion?

1☐ Never
2☐ Once per month or less
3☐ Two to four times a month
4☐ Two to three times a week
5☐ Four or more times a week

80 Have you ever felt you should cut down on your drinking?

1☐ Yes
2☐ No

81 Have people annoyed you by criticising your drinking?

1☐ Yes
2☐ No

82 Have you ever felt bad or guilty about your drinking?

1☐ Yes
2☐ No

83 Have you ever had a drink first thing in the morning to steady your nerves or get rid of a hangover (babalas)?

1☐ Yes
2☐ No

DRUG USE

84 Have you ever in your life used any of the following drugs? Please tick all drugs that you have **ever** used:

1☐ Dagga/weed/marijuana/pot/hash
2☐ Poppers
3☐ Cocaine
4☐ Wunga
5☐ Crack
6☐ Khat
7☐ Tik (crystal meth)
8☐ Mandrax/buttons
9☐ Psychedelics/LSD/mushrooms/other hallucinogens
10☐ Heroin/ other opiates
11☐ Uppers, speed (amphetamines)
12☐ Any other drugs
13☐ I never used any drugs → **IF YOU NEVER USED ANY DRUGS CONTINUE WITH QUESTION 90.**

85 Please indicate for each of the following drugs whether you have used them **in the past three months**:

- 1☐ Dagga/weed/marijuana/pot/hash
- 2☐ Poppers
- 3☐ Cocaine
- 4☐ Wunga
- 5☐ Crack
- 6☐ Khat
- 7☐ Tik (crystal meth)
- 8☐ Mandrax/buttons
- 9☐ Psychedelics/LSD/mushrooms/other hallucinogens
- 10☐ Heroin/ other opiates
- 11☐ Uppers, speed (amphetamines)
- 12☐ Any other drugs
- 13☐ I never used any drugs

86 Have you ever used a needle to inject drugs such as heroin (not prescribed medications) into your body?

- 1☐ Yes
- 2☐ No, never **IF "NEVER" CONTINUE WITH QUESTION 89.**

87 **IF YES**: Have you used a needle to inject drugs into your body **in the past year**?

- 1☐ Yes
- 2☐ No, never

88 Think about the **last time** you injected drugs. Did you use a needle that had previously been used by someone else?

- 1☐ Yes
- 2☐ No

89 When you look at your drug use behavior **in the past three months**, how do you assess the chances that you might have become infected with HIV?

- 1☐ No chance at all
- 2☐ A minor chance
- 3☐ A reasonable chance
- 4☐ A major chance

PREVENTING HIV

90 There are several ways in which it is possible to prevent transmission of HIV. Indicate for each of the following questions whether you think it is true or not. People can protect themselves from HIV by:

	True	False	Don't know	
a)	1☐	2☐	3☐	Having a good diet
b)	1☐	2☐	3☐	Staying with one faithful partner
c)	1☐	2☐	3☐	Avoiding public toilets
d)	1☐	2☐	3☐	Using condoms during sexual intercourse
e)	1☐	2☐	3☐	Avoiding touching a person who has AIDS
f)	1☐	2☐	3☐	Avoiding sharing food with a person who has AIDS
g)	1☐	2☐	3☐	Avoiding being bitten by mosquitoes or similar insects
h)	1☐	2☐	3☐	Making sure any injection they have is done with a clean needle
i)	1☐	2☐	3☐	Avoid sharing razor blades
j)	1☐	2☐	3☐	Only having sex with persons of the same sex

91 There are several things that women who have sex with other women can use to avoid transmission of HIV, such as latex gloves, dental dams, plastic wrap, and finger cots. Did you know that women can use such things when they have sex with other women to protect themselves?

- 1☐ Yes, I knew
- 2☐ No, I did not know

92 How do you or would you feel about using these things (latex gloves, dental dams, plastic wrap, and finger cots) to prevent HIV transmission?

- 1☐ Very comfortable
- 2☐ Comfortable
- 3☐ Neutral
- 4☐ Uncomfortable
- 5☐ Very uncomfortable

93 Do you know where you would be able to get things like latex gloves, dental dams, saran wrap, and finger cots that can be used to prevent HIV transmission?

- 1☐ I don't know
- 2☐ I know
- 3☐ These things are not available where I live

94 Another way to prevent HIV transmission is by not having sex when you or your female partner has your/her period (menstruation). How do you or would you feel about not having sex when you or your female partner has your/her period?

- 1☐ Very comfortable
- 2☐ Comfortable
- 3☐ Neutral
- 4☐ Uncomfortable
- 5☐ Very uncomfortable

95 Do you agree or disagree with the following statement: "Most women who are important to me think I should avoid sex when I or my female partner has my/her period?"

- 1☐ Completely disagree
- 2☐ Disagree
- 3☐ Agree
- 4☐ Completely agree
- 5☐ Don't know

96 How difficult or easy would it be for you to avoid sex when you or your female partner have your/her period?

- 1☐ Very difficult
- 2☐ Difficult
- 3☐ Easy
- 4☐ Very easy
- 5☐ Don't know

97 Which of the following statements do you think is **most** true (tick one)?

- 1☐ There is no way to treat people with HIV
- 2☐ There is medication that can completely cure AIDS
- 3☐ AIDS medication can slow the disease
- 4☐ AIDS medication only makes you sicker

98 If you would be infected with HIV, would you use HIV medication?

- 1☐ Definitely not
- 2☐ Maybe
- 3☐ Definitely yes

99 If you would be infected with HIV, would you consult a traditional healer?

- 1☐ Definitely not
- 2☐ Maybe
- 3☐ Definitely yes

SEXUAL HEALTH INFORMATION & CARE

100 If you have sex, you may also have a sexually transmitted infection (STI), along with subtle or noticeable STI symptoms. Lesbian or straight, married or single, you're vulnerable to STIs and STI symptoms, whatever sex you engage in. Please indicate whether you have had any of the symptoms in the past year:

	Yes	No	
a)	1☐	2☐	Strong vaginal smell (e.g., fishy odor)
b)	1☐	2☐	Vaginal itching or irritation
c)	1☐	2☐	Light vaginal bleeding (not during menstruation)
d)	1☐	2☐	Frequent urination
e)	1☐	2☐	Pain or burning sensation when urinating
f)	1☐	2☐	Lower abdominal pain
g)	1☐	2☐	Thick, cloudy or bloody discharge from the vagina
h)	1☐	2☐	Greenish yellow, possibly frothy vaginal discharge
i)	1☐	2☐	Pain during sexual intercourse

101 If you were to experience any of the above symptoms, what would you do? Please tick all that apply.

1☐ Wait and see if they go away
2☐ Wait until it gets serious and then see a doctor
3☐ Ask other women whether they know what the problem is
4☐ Go to a chemist or pharmacy
5☐ Get advice from a traditional healer
6☐ See a medical doctor
7☐ Get a test at a public clinic/hospital
8☐ Inform your sexual partner
9☐ Stop having sex when you had the symptoms
10☐ Use a condom (or other barrier) when having sex during the time you had the symptoms
11☐ Other; please specify _______________

102 In the past year, has a medical doctor or a nurse ever said to you that you had a sexually transmitted disease or infection such as Hepatitis B and C, Herpes, Gonorrhea, HPV, Chlamydia, Syphilis?

1☐ Yes
2☐ No

103 The following questions are about HIV testing. Please remember that this information is completely confidential. Do you think you have HIV?

1☐ No
2☐ Yes
3☐ Don't know

104 Have you ever been tested for HIV?

1☐ Yes
2☐ No

105 **IF YOU HAVE NEVER BEEN TESTED FOR HIV:** What are your main reasons for not getting tested? Please tick each reason that applies to you and continue with question 111.

1☐ Fear of finding out the results
2☐ I don't think I am at risk
3☐ I always use protection
4☐ Cost of having the test done
5☐ I don't know places where I can go to have the test done
6☐ I fear being judged/discriminated against when asking to have the test done
7☐ I am embarrassed that people will think I am gay/lesbian or bisexual
8☐ Other; please specify _______________

106 **IF YOU HAVE BEEN TESTED FOR HIV:** How many times in total have you been tested for HIV?

________ times

107 **IF YOU HAVE BEEN TESTED FOR HIV:** What led you to take the test? Please tick each reason that applies to you.

- 1☐ I wanted to know if I was infected
- 2☐ I was having some symptoms
- 3☐ I had had unprotected sex
- 4☐ I found out one of my sexual partners has HIV
- 5☐ I went to accompany a friend and decided to have it done
- 6☐ It was part of a medical examination
- 7☐ It was required by my employer
- 8☐ For insurance purposes
- 9☐ Other; please specify _______________

108 **IF YOU HAVE BEEN TESTED FOR HIV:** Do you feel positive or negative about the way you were treated when you got tested for HIV?

- 1☐ Very positive
- 2☐ Positive
- 3☐ Neutral
- 4☐ Negative
- 5☐ Very negative

109 **IF YOU HAVE BEEN TESTED FOR HIV:** When you were tested, did you receive information about safer sex for lesbian women and other women who have sex with women?

- 1☐ No
- 2☐ Yes

110 **IF YOU HAVE BEEN TESTED FOR HIV:** Have you ever tested HIV positive?

- 1☐ No
- 2☐ Yes

111 How much information about HIV/AIDS have you obtained from each of the following sources:

A lot	Some	None	
1☐	2☐	3☐	TV
1☐	2☐	3☐	Radio
1☐	2☐	3☐	Newspapers
1☐	2☐	3☐	Books and magazines
1☐	2☐	3☐	Pamphlets
1☐	2☐	3☐	Internet
1☐	2☐	3☐	Health Workers
1☐	2☐	3☐	Friends
1☐	2☐	3☐	Relatives
1☐	2☐	3☐	Partner(s)

112 Have you ever received STI/HIV information specifically for women who have sex with women?

- 1☐ No
- 2☐ Yes

113 If yes, how was that information provided to you? (Tick all that apply)

- 1☐ TV
- 2☐ Radio
- 3☐ Newspapers
- 4☐ Books and magazines
- 5☐ Pamphlets/flyers
- 6☐ Internet
- 7☐ Health Workers
- 8☐ Friends
- 9☐ Relatives
- 10☐ Partner(s)
- 11☐ LGBT or AIDS service organisation
- 12☐ Other; please specify _______________

114 From which source or sources would you prefer to learn about HIV, safe sex practices, and testing for women? Tick all that apply.

- 1☐ TV
- 2☐ Radio
- 3☐ Newspapers
- 4☐ Books and magazines
- 5☐ Pamphlets/flyers
- 6☐ Internet
- 7☐ Health Workers
- 8☐ Friends
- 9☐ Relatives
- 10☐ Partner(s)
- 11☐ LGBT or AIDS service organisation
- 12☐ Other; please specify _______________

115 The following questions are about your health care. Where do you usually go to get your health care?

- 1☐ Private doctor
- 2☐ Local clinic (HIV clinic/Health clinic)
- 3☐ Public hospital
- 4☐ Faith based services
- 5☐ Traditional healer
- 6☐ LGBT Clinic
- 7☐ A non-governmental organisation

116 In general, how hard do you try to keep your sexual orientation secret from medical doctors and nurses?

- 1☐ I try very hard to keep it secret
- 2☐ I try somewhat hard to keep it secret
- 3☐ I don't try to keep it secret
- 4☐ I openly talk about it with medical doctors and nurses

117 If you were to tell a medical doctor or nurse about your sexual orientation, what impact do you think it would have on the care you will receive/the way you are treated?

- 1☐ Very negative
- 2☐ Negative
- 3☐ Positive
- 4☐ Very positive
- 5☐ No impact

118 Would you tell a medical doctor or nurse about your sexual orientation?

- 1☐ Yes, I would tell them on my own
- 2☐ Yes, but only if asked
- 3☐ No, but I wish I could
- 4☐ No, I prefer not to
- 5☐ No, there is no reason to

119 There are several reasons why women don't seek health care services. Please tick the reasons that apply or would apply to you.

- 1☐ I am not aware that such services exist where I live
- 2☐ I don't have transport to get to the places where these services are provided
- 3☐ The services that are provided are not sensitive to the needs of lesbian women and other women who have sex with women
- 4☐ The organization (s) providing the services might not or does not respect my privacy
- 5☐ The organization(s) providing the services do not speak my primary language
- 6☐ I do not have medical aid
- 7☐ I cannot afford to receive the services
- 8☐ The organizations providing the services are only open during times that are inconvenient for me
- 9☐ Other; please specify _______________

GENDER AND SEXUAL IDENTITY

120 How do you currently see yourself in terms of your gender?

- 1☐ Woman in a woman's body
- 2☐ Man in a woman's body
- 3☐ Woman in a man's body
- 4☐ Other; please specify _______________

121 Indicate on a scale from 1 (not at all) to 5 (extremely) how masculine and feminine you think you are. Masculine refers to persons who feel, look and act like "real" men or in a manner which most people think that men should be like. Feminine is the opposite of masculine and refers to what usually is expected from women. People, men or women, who look and behave like "real" women are called feminine.

	**Not at all**	**A little**	**Some-what**	**Very much**	**Extremely**
a) In general, how feminine do you think you are?	1☐	2☐	3☐	4☐	5☐
b) How feminine do you act, appear and come across to others?	1☐	2☐	3☐	4☐	5☐
c) How feminine is your personality?	1☐	2☐	3☐	4☐	5☐
d) In general, how masculine do you think you are?	1☐	2☐	3☐	4☐	5☐
e) How masculine do you act, appear and come across to others?	1☐	2☐	3☐	4☐	5☐
f) How masculine is your personality?	1☐	2☐	3☐	4☐	5☐

122 Please indicate to what extent you agree or disagree with each of the following statements:

	**Disagree strongly**	**Disagree**	**Agree**	**Agree strongly**
a) Sometimes I dislike myself for being a person who has (or wants) sex with people of the same sex	1☐	2☐	3☐	4☐
b) I wish I were only sexually attracted to the opposite sex	1☐	2☐	3☐	4☐
c) I am not proud of myself for being sexually attracted to people of the same sex	1☐	2☐	3☐	4☐
d) I feel that being attracted to people of the same sex is a personal weakness of mine	1☐	2☐	3☐	4☐
e) If someone offered me the chance to be completely heterosexual, I would accept the offer	1☐	2☐	3☐	4☐
f) Whenever I think about having sex with someone of the same sex, I feel bad about myself	1☐	2☐	3☐	4☐

123 How many people in your family know that you are sexually attracted to persons of the same sex?

- 1☐ None of them
- 2☐ Some of them
- 3☐ All of them

124 How many friends and/or colleagues know that you are sexually attracted to persons of the same sex?

- 1☐ None of them
- 2☐ Some of them
- 3☐ All of them

125 In general, how hard do you try to keep your sexual orientation secret **from your family**?

- 1☐ I try very hard to keep it secret
- 2☐ I try somewhat hard to keep it secret
- 3☐ I don't try to keep it secret
- 4☐ I openly talk about it with my family

126 In general, how hard do you try to keep your sexual orientation secret **from your friends**?

- 1☐ I try very hard to keep it secret
- 2☐ I try somewhat hard to keep it secret
- 3☐ I don't try to keep it secret
- 4☐ I openly talk about it with my friends

127 In general, how hard do you try to keep your sexual orientation secret **at work, school, college** or **university**?

- 1☐ I try very hard to keep it secret
- 2☐ I try somewhat hard to keep it secret
- 3☐ I don't try to keep it secret
- 4☐ I openly talk about it with others at work, school, college or university

128 Below are some statements about how you could feel about being part of the general community where you currently are living. Please indicate whether you agree or disagree with these statements.

	Disagree strongly	Disagree	Agree	Agree strongly
a) Where I live, people accept me	1☐	2☐	3☐	4☐
b) I feel misunderstood where I live	1☐	2☐	3☐	4☐
c) I am part of the community where I live	1☐	2☐	3☐	4☐
d) I feel like an outsider where I live	1☐	2☐	3☐	4☐

129 Below are some statements about how you could feel about being part of the LGBT community in South Africa. Please indicate whether you agree or disagree with these statements.

	Disagree strongly	Disagree	Agree	Agree strongly
a) Other LGBT people accept me	1☐	2☐	3☐	4☐
b) I feel like an outsider in the LGBT community	1☐	2☐	3☐	4☐
c) I feel part of the LGBT community	1☐	2☐	3☐	4☐
d) I feel misunderstood in the LGBT community	1☐	2☐	3☐	4☐

EMOTIONAL HEALTH

130 The 10 items below refer to how you have felt and behaved **over the last 4 weeks**. Please indicate for each item how often you felt or behaved that way.

	None of the time	A little of the time	Some of the time	Most of the time	All of the time
a) In the past 4 weeks, how often did you feel tired for no good reason?	1☐	2☐	3☐	4☐	5☐
b) In the past 4 weeks, how often did you feel nervous?	1☐	2☐	3☐	4☐	5☐
c) In the past 4 weeks, how often did you feel so nervous that nothing could calm you down?	1☐	2☐	3☐	4☐	5☐
d) In the past 4 weeks, how often did you feel hopeless?	1☐	2☐	3☐	4☐	5☐
e) In the past 4 weeks, how often did you feel restless or fidgety?	1☐	2☐	3☐	4☐	5☐
f) In the past 4 weeks, how often did you feel so restless you could not sit still?	1☐	2☐	3☐	4☐	5☐
g) In the past 4 weeks, how often did you feel depressed?	1☐	2☐	3☐	4☐	5☐
h) In the past 4 weeks, how often did you feel that everything was an effort?	1☐	2☐	3☐	4☐	5☐
i) In the past 4 weeks, how often did you feel so sad that nothing could cheer you up?	1☐	2☐	3☐	4☐	5☐
j) In the past 4 weeks, how often did you feel worthless?	1☐	2☐	3☐	4☐	5☐

COMPLETING THIS QUESTIONNAIRE

131 Did you find it difficult or easy to understand the questions?

- 1☐ Very easy
- 2☐ Easy
- 3☐ Difficult
- 4☐ Very difficult

132 Did you fill out this questionnaire on your own or did somebody help you?

- 1☐ On my own
- 2☐ Somebody helped me

133 Did you experience any problems filling out this questionnaire?

- 1☐ No problems
- 2☐ Few problems
- 3☐ Many problems
